# Identifying disease genes by integrating multiple data sources

**DOI:** 10.1186/1755-8794-7-S2-S2

**Published:** 2014-10-22

**Authors:** Bolin Chen, Jianxin Wang, Min Li, Fang-Xiang Wu

**Affiliations:** 1 Division of Biomedical Engineering, University of Saskatchewan, 57 Campus Dr., S7N 5A9, Saskatoon, Canada; 2 School of Information Science and Engineering, Central South University, 410083, Changsha, P.R. China; 3 Department of Mechanical Engineering, University of Saskatchewan, 57 Campus Dr., S7N 5A9, Saskatoon, Canada

**Keywords:** disease gene, data integration, Markov random field, Bayesian analysis

## Abstract

**Background:**

Now multiple types of data are available for identifying disease genes. Those data include gene-disease associations, disease phenotype similarities, protein-protein interactions, pathways, gene expression profiles, *etc*.. It is believed that integrating different kinds of biological data is an effective method to identify disease genes.

**Results:**

In this paper, we propose a multiple data integration method based on the theory of Markov random field (MRF) and the method of Bayesian analysis for identifying human disease genes. The proposed method is not only flexible in easily incorporating different kinds of data, but also reliable in predicting candidate disease genes.

**Conclusions:**

Numerical experiments are carried out by integrating known gene-disease associations, protein complexes, protein-protein interactions, pathways and gene expression profiles. Predictions are evaluated by the leave-one-out method. The proposed method achieves an AUC score of 0.743 when integrating all those biological data in our experiments.

## Background

Many human genetic diseases or disorders are resulted from mutations of multiple genes [1]. The identification of those disease genes is not only important in understanding genetic disease mechanisms, but is also helpful in developing new methods in diagnostics and therapeutics [2].

Genes associated with similar disorders are often functionally related, supporting the existence of distinct disease-specific functional modules [3-5]. A "guilt-by-association" [6] assumption is often used by various algorithms to identify disease genes. If a gene is ranked as "close" to known disease genes, it would be likely regarded as related to the same disease. The principle is largely supported by many biological data sources, such as protein-protein interactions (PPIs) [7-11], pathways [12-15], gene expression profiles [16-18], *etc*.. Lage et al. [19] rank disease genes from a constructed phenome-interactome network by using PPIs and phenotype similarities. Wu et al. [5] develop a tool called CIPHER to predict disease genes based on a global concordance between a PPI network and a phenotype network. Hwang et al. [20] use a similar coherence score between a gene network and a phenotype network. Vanunu et al. [21] design a method called PRINCE that predicts disease genes and protein complexes associated with diseases at the same time. Li et al. [22] analyze human disease and disease relationships from a pathway-based point of view. Ma et al. [23] employs the Markov Random Field (MRF) theory to prioritize genes associated with a specific phenotype or trait by using gene expression profiles and PPI data.

Multiple data integration is another commonly used methodology that collects evidences of gene disease associations from different data sources. Köhler et al. [24] propose a random walk with restart (RWR) algorithm that predicts disease genes by using *a mixed PPI network*. Zhang et al. [25] develop a Bayesian regression approach to explain similarities between disease phenotypes by using diffusion kernels of one or several PPI networks. Chen et al. [26] define a data integration rank (DIR) score by taking a *max *instead of *average *to capture the most informative evidence among a set of integrated data sources. The DIR algorithm potentially yields better performance than many other data integration methods [26].

However, challenges still exist because of the following reasons. Firstly, there are many levels of controls along paths from genotypes to phenotypes [26]. Genes have to be transcribed and then be translated into proteins, and proteins interact with many other molecules to perform cellular functions [26-28], resulting in the complex relationship between genotypes and phenotypes [29]. Secondly, different biological data are heterogeneous. They describe relationships of molecular entities in various levels. No widely acceptable criterion is available to standardize them into the same scale. An inappropriate integration method combines noise as well, which often decreases the prediction accuracy. Thirdly, many "guilt-by-association" methods only take edges of a candidate gene with known disease genes into account, ignoring edges of the gene with many other vertices in a biological network. They ignore the fact that the biological network, let's say a PPI network or a gene co-expression network, is built independently for describing a specific biological relationship of proteins or genes. It may have no direct relationship with gene disease associations.

In this paper, we introduce a multiple data integration method for disease gene identifications, which considers comprehensive characters of a set of heterogeneous datasets to capture the complex relationship between genotypes and phenotypes. The method is based on the theory of MRF and the method of Bayesian analysis. Two previous algorithms of Deng et al. [30] and Ma et al. [23] have been proposed to integrating multiple datasets by using the MRF theory for yeast protein function predictions. Their method cannot be directly employed to identify human disease genes. Predictions of the method of Deng et al. [30] become unreliable due to the following scale problem. Human genome consists of around 21,000 genes [31], while most diseases are associated by mutations of only a few genes. Even merging similar diseases into classes, the associated genes of individual disease classes is still not enough to estimate parameters correctly by using Deng's method. The method by Ma et al. [23] mainly uses gene expression profiles to group genes with similar characters. PPI data are only employed to calibrate predictions. It is not clear how to integrate more kinds of biological data by using their method. In paper [32], we have developed a basic modified MRF model for human disease gene prioritization. In this study, we will further improve it by introducing a new parameter estimation strategy and a new Gibbs sampling strategy. The improved MRF algorithm is not only stable in terms of parameter estimation, but also reliable in terms of its prediction accuracy.

## Methods

In this paper, we first briefly describe how the problem is formulated as a Bayesian labelling problem. The labelling configration assumes to follow a Gibbs distribution. After that, a MRF model is introduced to solve this problem by integrating multiple kinds of biological data, including known gene-disease associations, protein complexes, PPIs, pathways and gene expression profiles.

### The Bayesian labelling problem

Let *L *= {*L*_1_*, L*_2_, ..., *L_k_*} be a set of *k *labels and *S *= {*S*_1_*, S*_2_, ... *, S_r_*} be a set of *r *sites. A *labelling problem *[33] is defined as assigning each site *Si *with a label in *L*.

Let *F *= {*F*_1_*, F*_2_, ... *, F_r_*} be a family of random variables defined on *S*, in which each random variable *F_i _*takes value *f_i _*of *L*. We use the notation *F *= *f *to represent the joint event that {*F*_1 _= *f*_1_, ... *, F_r _*= *f_r_*}, where *f *= {*f*_1_, ... *, f_r_*} is called a *configuration *of *F*. The set of all configurations is denoted as  F.

The relationship of sites is determined by a neighborhood system $N=\left\{N_{i}|\forall_{i}\in S\right\}$, where *N_i _*is the set of sites neighboring *i*.

A family of random variables *F *is said to be a MRF on *S *w.r.t. *N *if and only if the following two conditions are satisfied:

1 Positivity: P(f)>0,∀f∈F,

2 Markovianity: $P\left(f_{i}|F_{S\backslash \left\{i\right\}}\right)=P\left(f_{i}|f_{N_{i}}\right).$

The Markovianity indicates that the probability of a local event *f_i _*conditioned on all other events is equivalent to that conditioned on only events of its neighbors. Hence, the joint probability *P*(*f*) of the random field can be uniquely determined by local conditional probabilities.

Let ***r ***be an observation of *F *. Suppose we know both the prior probability distribution *P *(*f*) of configuration *f *and the conditional probability distribution *P *(***r**|f*) of the observation ***r ***given the configuration *f*. The best estimation of *f *is the one maxizing a posteriori probability (MAP), which is

(1) P(f|r)=P(r|f)P(f)/P(r)

where *P*(***r***) is the probability that we get the observation ***r***.

The *Bayesian labelling problem *[33] is that given a set of observation ***r***, find the MAP configuration of labelling $f^{*}={\text{arg max}}_{f\in F}P\left(f|r\right)$. Here, as *P *(***r***) is not a function of *f *, it does not affect the MAP estimation of *f*.

### Gibbs distribution in MRF

It is usually hard to specify a prior probability of a MRF for a real problem. Fortunately, the Hammersley-Clifford theorem [34] provides a solution for this. According to the theorem, *F *is a MRF on *S *w.r.t.  N if and only if the probability distribution of *P *(*F *= *f*) of the configuration is a Gibbs distribution w.r.t.  N. The Gibbs distribution has a form of

(2) $$P\left(f\right)=Z^{-1}\cdot e^{-U\left(f\right)/T},$$

where $Z=\sum_{f\in F}e^{-U\left(f\right)/T}$ is a normalizing constant, *T *is a global control constant that is often assumed to be 1, and *U*(*f*) is the energy function calculated as follows

(3) $$\begin{array}{c} U\left(f\right)=\underset{c\in C}{\sum }V_{c}\left(f\right)\\ =\underset{\left\{i\right\}\in C_{1}}{\sum }V_{1}\left(f_{i}\right)+\underset{\{\left(i,j)\right\}\in C_{2}}{\sum }V_{2}\left(f_{i},f_{j}\right)+R_{n}\left(f\right), \end{array}$$

where *V_i_*(*f*) is the energy potential of *C_i _*(the set of *i^th ^*order cliques) in the neighborhood system  N, *R_n_*(*f*) represents those higher order terms. A special case of MRF is the Ising model that only considers up to the second order of cliques [35].

Given a configuration *f*, let the conditional probability distribution of observation r have the same exponential form

(4) $$P\left(r|f\right)=Z_{r}^{-1}\cdot e^{-U\left(r|f\right)}.$$

Then the posterior probability of the Gibbs distribution has form

(5) $$P\left(f|r\right)=Z_{E}^{-1}\cdot e^{-U\left(f|r\right)},$$

where the posterior energy is [33]

(6) U(f|r)=U(f)+U(f|r).

Based on this, suppose the collection of whole human genes *G *= {*g*_1_, *g*_2_, ..., *g_N_*} is the site set, and {1, 0} is the label set, where 1 represents a gene is a disease gene and 0 otherwise. The problem of human disease gene identification is actually to find the best configuration of *G *according to what is currently known about human diseases.

### The MRF model for identifying human disease genes

Suppose human genome consists of a set of *N *genes *G *= {*g*_1_, *g*_2_, ..., *g_N_*}. Some of them are already known to be associated with genetic diseases, while associations of most other genes are still not known. Without loss of generality, let *g*_1_; *g*_2_, ..., *g_n _*be genes that have not yet been known to be associated with genetic diseases, and *g_n+1_, g_n+2_*, ..., *g_n+m _*be currently known disease genes. Obviously, we have *N *= *n *+ *m*. Let {*D*_1_*, D*_2_, ..., *D_M _*} be a set of human diseases, where *D_i _*consists of the set of genes that are already known associated with the *i^th ^*disease.

For a specific disease, let *X *= (*X*_1_*, X*_2_, ..., *X_n+m_*) be the random variables defined on all genes, where *X_i _*= 1 represents gene *g_i _*to be a associated gene of the disease and *X_i _*= 0 otherwise.

Consider those individual genes. Let (*π*_1_*, π*_2_, ..., *π_n+m_*) be a set of probabilities, where *π_i _*represents the probability that *X_i _*= 1. Let *x *= (*x*_1_*, x*_2_, ..., *x_n+m_*) be observations of *X*. The probability distribution of configuration *x *is proportional to

(7) $$\begin{array}{c} \overset{n+m}{\underset{i=1}{\prod }}\pi_{i}=\overset{n+m}{\underset{i=1}{\prod }}\pi_{i}^{x_{i}}{\left(1-\pi_{i}\right)}^{1-x_{i}}\\ =\overset{n+m}{\underset{i=1}{\prod }}{\left(\frac{\pi_{\iota }}{1-\pi_{i}}\right)}^{x_{i}}\left(1-\pi_{i}\right)\\ =\text{exp}\;\left[\overset{n+m}{\underset{i=1}{\sum }}\alpha_{i}x_{i}+\overset{n+m}{\underset{i=1}{\sum }}\text{log}\left(1-\pi_{i}\right)\right]\propto \text{ exp}\overset{n+m}{\underset{i=1}{\sum }}\alpha_{i}x_{i} \end{array}$$

where $\alpha_{i}=\text{log}\frac{\pi_{i}}{1-\pi_{i}}$, and $\sum_{i=1}^{n+m}\text{log}\left(1-\pi_{i}\right)$ is a constant.

Next, consider pairwise relationships between genes. Suppose we have *K *biological networks *H *= (*H*^1^, ..., *H^K^*), where vertices represent genes. Given a *H^k^*, edges of *H^k ^*represent a specific kind of biological relationship between those genes. Let *x *be the observation labels of *X*. According to *x*, edges of *H^k ^*can be classified into three categories: (1) edges that between two 1-labelled vertices, (2) edges that between a 1-labelled vertex and a 0-labelled vertex, and (3) edges that between two 0-labelled vertices. Let $N_{11}^{k},N_{10}^{k}$ and $N_{00}^{k}$ denote the number of edges in each category of *G^k ^*respectively. Then

(8) $$N_{11}^{k}=\underset{\left\{\left(i,j\right)\right\}\in E\left(H^{k}\right)}{\sum }x_{i}x_{j},$$

(9) $$N_{10}^{k}=\underset{\left\{\left(i,j\right)\right\}\in E\left(H^{k}\right)}{\sum }\left(1-x_{i}\right)x_{j}+x_{i}\left(1-x_{j}\right),$$

(10) $$N_{00}^{k}=\underset{\left\{\left(i,j\right)\right\}\in E\left(H^{k}\right)}{\sum }\left(1-x_{i}\right)\left(1-x_{j}\right).$$

The probability that we have such a kind of biological network *H^k ^*conditional on those observed labels *x *follows as

(11) $$P\left(H^{k}|x,\theta^{k}\right)\propto e^{\beta^{k}}N_{10}^{k}+\gamma^{k}N_{11}^{k}+K^{k}N_{00}^{k},$$

where *θ^k ^*= (*β^k^, γ^k^, κ^k^*) are weights of these three kinds of edges for *H^k^*. One of three parameters in *θ^k ^*is redundant. Without loss of generality, let *κ^k ^*= 1. Similarly, for *K *biological networks, the probability that we observe them conditional on the observed labels follows as

(12) $$P\left(H^{1},\ldots ,H^{K}|x,\theta^{1},\ldots \theta^{K}\right)\propto \overset{K}{\underset{k=1}{\sum }}e^{\beta^{k}}N_{10}^{k}+\gamma^{k}N_{11}^{k}+N_{00}^{k}.$$

Based on the Ising model, the energy function can be written in terms of *x *as

(13) $$U\left(x|0\right)=-\overset{n+m}{\underset{i=1}{\sum }}\alpha_{i}x_{i}-\overset{K}{\underset{k=1}{\sum }}\left(\beta^{k}N_{10}^{k}+\gamma^{k}N_{11}^{k}+N_{00}^{k}\right)$$

where *θ *= (*α_i_, β*^1^*, γ*^1^, ..., *β^K ^, γ^K^*) are parameters. In the terminology of MRF [30], *U *(*x|θ*) defines a Gibbs distribution of the entire networks

(14) $$P\left(x|\theta \right)=\frac{1}{Z\left(\theta \right)}\times e^{-U\left(x|\theta \right)},$$

where *Z*(*θ*) is the normalized constant that is calculated by summing over all configurations *χ*:

$$Z\left(\theta \right)=\underset{x\in \chi }{\sum }e^{-U\left(x|\theta \right)}.$$

### The Gibbs sampling

The Gibbs distribution (14) gives a prior probability distribution of the configuration for all genes. In the study of identifying human disease genes, the objective is to find the posterior probability of *X*_1_, *X*_2_, · · ·, *X_n _*conditional on known disease genes

$$P\left(X_{1},X_{2},\cdots \;,X_{n}|X_{n+1},X_{n+2},\cdots \;,X_{n+m}\right).$$

To achieve this, consider the following posterior probability distribution of an individual gene *X_i_*

$$P\left(X_{i}=1|X_{\left[-i\right]},\theta \right)$$

where *X*_[*−i*] _= (*X*_1_, · · ·, *X_i−1_, X_i+1_* , · · ·, *X_n+m_*) represents labels of all other genes except *X_i_, θ *are parameters. According to the Bayes' theorem [36] and the Gibbs distribution (14), we have

(15) $$\begin{array}{c} P\left(X_{i}=1|X_{\left[-i\right]},\;\theta \right)\\ \;=\frac{P\left(X_{i}=1,X_{\left[-i\right]}|\theta \right)}{P\left(X_{i}=1,X_{\left[-i\right]}|\theta \right)+P\left(X_{i}=0,X_{\left[-i\right]}|\theta \right)}\\ \;=\frac{e^{-U(X_{i}=1,X_{\left[-i\right]}|\theta )}}{e^{-U\left(X_{i}=1,X_{\left[-i\right]}|\theta \right)}+e^{-U\left(X_{i}=0,X_{\left[-i\right]}|\theta \right)}}\\ \;=\frac{e^{T\left(i\right)}}{e^{T\left(i\right)}+1}. \end{array}$$

where

(16) $$\begin{array}{c} U\left(X_{i}=1,\;X_{\left[-i\right]}|\theta \right)=U\left(X_{\left[-i\right]}|\theta \right)-\alpha_{i}\\ \;\;\;\;\;\;\;\;\;-\overset{K}{\underset{k=1}{\sum }}\left(\beta^{k}M_{0}^{k}-\gamma^{k}M_{1}^{k}\right), \end{array}$$

(17) $$\begin{array}{c} U\left(X_{i}=0,\;X_{\left[-i\right]}|\theta \right)=U\left(X_{\left[-i\right]}|\theta \right)\\ \;\;\;\;\;\;\;\;\;-\overset{K}{\underset{k=1}{\sum }}\left(\beta^{k}M_{1}^{k}-M_{0}^{k}\right), \end{array}$$

the according to equation (13), and

(18) $$\begin{array}{c} T\left(i\right)=-U\left(X_{i}=1,\;X_{\left[-i\right]}|\theta \right)+U\left(X_{i}=0,\;X_{\left[-i\right]}|\theta \right)\\ =\alpha_{i}+\overset{K}{\underset{k=1}{\sum }}\left[\left(\beta^{k}-1\right)M_{0}^{k}+\left(\gamma^{k}-\beta^{k}\right)M_{1}^{k}\right]. \end{array}$$

Here $M_{0}^{k}$ and $M_{1}^{k}$ are the number of neighbors of the gene $g_{i}$ labelled with 0 and 1 on network *H^k ^, k *= 1, ..., *K*, respectively.

Equation (15) provides a method to update the label *X_i _*according to all other labels. Suppose parameters *θ *= (*α_i_, β*^1^*, γ*^1^, ..., *β^K^, γ^K^*) of the model are given, together with prior observed labels of all genes. Using equation (15), we can update labels for all unknown genes. Repeating this procedure a number of times until all posterior probabilities of labels are stabilized. This is the essential procedure of the Gibbs sampling.

### Parameter estimation

In practice, we do not know parameters of the model and they need to be estimated according to those known informations. Ideally, the maximum likelihood estimation (MLE) method is a good choice to estimate *θ *in equation (14). However, the normalizing part *Z*(*θ*) is also a function of *θ*, which is the main difficulty for using the MLE method directly. Deng et al. [30] using a pseudo-likelihood method to estimate parameters in the MRF model. Specifically, the following pseudo-likelihood function is derived from equation (15), which is

(19) $$\text{log}\frac{P\left(X_{i}=1|X_{\left[-i\right]},\theta \right)}{1-P\left(X_{i}=1|X_{\left[-i\right]},\theta \right)}=T\left(i\right)$$

The parameter estimation can be done by a *binary logistic regression*, where dependent variables in equation (19) are categorical labels and independent variables are $M_{0}^{1},\;M_{1}^{1},\ldots ,M_{0}^{K},\;M_{1}^{K}$ of the *K *biological networks. The standard MATLAB function *glmf it*() can be employed to perform such binary logistic regression.

The pseudo-likelihood method used by Deng et al. [30] is valuable. However, there is an important potential problem [32,37], which may result in unreasonable predictions with their original method. The parameter estimation of Deng et al. [30] is conducted on only known labelled vertices of biological networks. However, a known vertex with labelling 1 may have plenty of unknown vertices with labelling 0 in a biological network and vice versa. A neglect of those unknown vertices may result in inaccurate estimated parameters, which makes predictions problematic. This problem becomes serious with the increasing number of unknown vertices [37]. Kourmpetis et al. [37] alternatively introduce a Bayesian MRF model to estimate parameters and update labels at the same time. An adaptive Markov Chain Monte Carlo (MCMC) algorithm is employed to perform the estimation by using another scaling parameter, a *Z *matrix and a multivariate normal distribution.

In this study, we introduce a new method to simultaneously estimate parameters and update labels. Suppose a prior probability of *π_i _*for each unknown vertex is known. A set of prior labels of unknown vertices can be assigned according to this probability. Then the pseudo-likelihood parameter estimation method is performed on all labeled vertices, including those known labelled ones and those unknown prior labelled ones. Using these estimated parameters to update labels for all unknown vertices, and then using the updated labels to re-estimate parameters until both of them are stable. The step-by-step description of this procedure is given as follows.

1 Initialization:

Let *t *= 0, and initialize labels of all vertices $\left(X_{1}^{\left(0\right)},X_{2}^{\left(0\right)},\ldots ,X_{n+m}^{\left(t\right)}\right)$

2 Estimating parameters:

$$\theta^{\left(t\right)}⇐\left(X_{1}^{\left(t\right)},X_{2}^{\left(t\right)},\ldots ,X_{n+m}^{\left(t\right)}\right);$$

3 Gibbs sampling:

$$\begin{array}{c} X_{1}^{\left(t+1\right)}⇐\left(\theta^{\left(t\right)},\;X_{2}^{\left(t\right)},\ldots ,X_{n+m}^{\left(t\right)}\right)\\ X_{2}^{\left(t+1\right)}⇐\left(\theta^{\left(t\right)},\;X_{1}^{\left(t+1\right)},\;X_{3}^{\left(t\right)},\ldots ,X_{n+m}^{\left(t\right)}\right)\\ X_{3}^{\left(t+1\right)}⇐\left(\theta^{\left(t\right)},\;X_{1}^{\left(t+1\right)},\;X_{2}^{\left(t+1\right)},\;X_{4}^{\left(t\right)},\ldots ,X_{n+m}^{\left(t\right)}\right)\\ \vdots \\ X_{n}^{\left(t+1\right)}⇐\left(\theta^{\left(t\right)},\;X_{1}^{\left(t+1\right)},\ldots ,X_{n-1}^{\left(t+1\right)},\;X_{n+1}^{\left(t\right)},\ldots ,X_{n+m}^{\left(t\right)}\right)\\ X_{n+1}^{\left(t+1\right)}⇐X_{n+1}^{\left(t\right)}\\ \vdots \\ X_{n+m}^{\left(t+1\right)}⇐X_{n+m}^{\left(t\right)} \end{array}$$

4 Let *t *= *t *+ 1, and go to 2, until stabilized.

During the Gibbs sampling procedure, a "burn-in period" and a "lag period" often need to be specified. The "burn-in period" is the period that a Markov process takes to become stabilized. Simulation results in this period are discarded to reduce the effect of initial prior probabilities. The "lag period" is the period that needs to reduce the dependence of the Markov process. The posterior probabilities in this period are estimated by averaging simulation results during individual lag steps.

In this study, the "burn-in period" takes 100 steps while the "lag period" takes 90 steps. Simulation results are averaged every 10 steps in the "lag period". There is 1000 steps in total for simulations. For convenience, predictions made by the original MRF model of Deng et al. [30] is denoted as "MRF-Deng", while predictions of our improved MRF method is denoted as "IMRF_1_" hereafter. A second improved MRF method is also given in the following by adding a new period at last in simulations, which is called "prediction period". It takes the average estimated parameters in the "lag period" as parameters and fixes them hereafter in simulations. The input probabilities of unknown vertices are also obtained by the average posterior probabilities in the "lag period". The Markov process runs another 100 steps in this period. The average posterior probabilities in the "prediction period" are outputted as final predictions, and predictions of this method is denoted as "IMRF_2_".

### Estimating a prior probability

Now, the only problem left is to estimate the prior probability of *π_i_*. Similarly as the method used in Deng et al. [30], we also estimate them according to known protein complexes. Since genes that encode proteins in a same complex tend to associated with similar diseases. For a gene *g_i _*that encodes protein in a complex,

(20) $${\hat{\pi }}_{i}=A/B$$

be the prior probability, where *A *is the number of disease genes for a specific disease in the complex, and *B *is the number of all disease genes in the complex. If a gene appears in multiple protein complexes, we use the maximum value as the prior probability for the gene.

For those genes that do not belong to any protein complex, let

(21) $${\hat{\pi }}_{i}=C/D$$

as the prior probability, where *C *is the number of all currently known disease genes for the specific disease, and *D *is the total number of genes in human genome.

### Data sources

The gene-disease association data are obtained from Goh et al. [3], which contain 1 284 disorders and 1 777 disease genes. These data are originally collected from the Morbid Map list of the Online Mendelian Inheritance in Man (OMIM) [38]. Disorders are manually classified into 22 primary disease classes, including a 'multiple' class and a 'unclassified' class. In this study, we consider only those disease classes that consist of at least 30 genes. We also exclude the 'multiple' class, the 'unclassified' class, the 'cancer' class and the 'neurological' class due to the class evidence and the class heterogeneity [3]. The final dataset consists of 815 genes in 12 disease classes.

The protein complex data are collected from the database of CORUM [39] and PCDq [40]. There are 1677 and 1103 protein complexes in the dataset that consist of at least two proteins, respectively. There are in total 3881 proteins in those protein complexes.

The PPI datasets are derived from the database of HPRD (Release 9) [9], BioGrid (Release 3.2.108) [10] and IntAct (downloaded on Jan 26, 2014) [11], respectively. Duplicated edges between the same pair of vertices and edges connecting to itself are deleted. Each dataset is processed independently, and three PPI networks are obtained finally. The HPRD PPI network consists of 9465 vertices and 37039 edges. The BioGrid PPI network consists of 15298 vertices and 127612 edges. The IntAct PPI network consists of 13449 vertices and 63825 edges.

The pathway datasets are obtained from the database of KEGG [12], Reactome [13], PharmGKB [14] and PIN [15], There are 280, 1469, 99 and 2679 pathways in datasets, respectively. There are in total 8614 proteins in those pathways. A pathway co-existing network is constructed by taking individual proteins/genes as vertices. Edges are constructed between two vertices, if they co-exist in any pathway.

The human gene expression profiles are obtained from BioGPS (GSE1133) [16,17], which contain 79 human tissues in duplicates, measured using the Affymetrix U133A array. Pairwise Pearson correlation coefficients (PCC) are calculated and a pair of genes are linked by an edge if the PCC value is larger than 0.5, similar to the method used in [3,26].

Hence, five biological networks are constructed by collecting data from various databases. All protein IDs are mapped onto the form of the gene symbol. In order to test the performance of multiple data integration of our methods, we select those genes that appears at least four times in the five networks. The final datasets consist of 7311 human genes, 815 out of which are known associated with 12 disease classes.

### Validation method and evaluation criteria

The accuracy of predictions is validated by the leaveone-out method. For each known disease gene with at least one annotated interaction partner in a biological network, we assume it is an unknown gene and predict its posterior probability by our proposed methods. We use the receiver operating characteristic (ROC) curve to show the relationship between the true positive rate and the false positive rate by varying the threshold for declaring positives. The area under the ROC curve (AUC) is also employed to show an overall measure of the performance. The negative control set consists of known disease genes that do not belong to current disease class, and they are also validated by using the leave-one-out method.

### Decision score and declaration of positives

One can directly use the posterior probabilities obtained by the Gibbs sampling to select candidate disease genes. The greater the probability is for a gene, the more likely it is to associated with specific disease. However, different disease classes consist of different numbers of known disease genes, and thus the prediction results may not be good if a global threshold is used for all classes. Hence, we propose to use a percentage as a decision score to generate the finial predictions. All the ROC curves and the AUC scores of our "IMRF_1_" and "IMRF_2_" method are calculated according to the decision score hereafter.

## Results and discussion

We first analyze the performance of the IMRF_1 _and IMRF_2 _algorithms in terms of stability and reliability, and then compare our method with the original MRF-Deng method [30], the RWR algorithm [24] and the DIR algorithm [26]. These three algorithms are selected elaborately.

Firstly, since ideas of our improved methods (IMRF_1 _and IMRF_2_) are initially inspired by the MRF-Deng method, the direct comparison illustrates how much improvement can be made results from our methods.

Secondly, we compare our methods with the RWR algorithm to show which manner of multiple data integration is better. The RWR algorithm is a typical data integration method that uses a mixed network, where vertices and edges of several biological networks are simply merged together, while our methods integrate different networks separately.

Finally, the DIR algorithm has a very good performance among multiple data integration methods, which also integrates different networks separately. It is the same with our methods in terms of the data integration method.

### Stability and reliability of MRF methods

We first investigate the stability and reliability MRF methods, by analyzing Markov processes of the IMRF_1 _method and the MRF-Deng method.

Parameters of the MRF-Deng method are estimated from subnetworks of known vertices. This is feasible to be used for predicting protein functions of yeast in [30], since each function class consists of at least hundreds known vertices, which is possible for estimating reasonable parameters.

**Figure 1:**
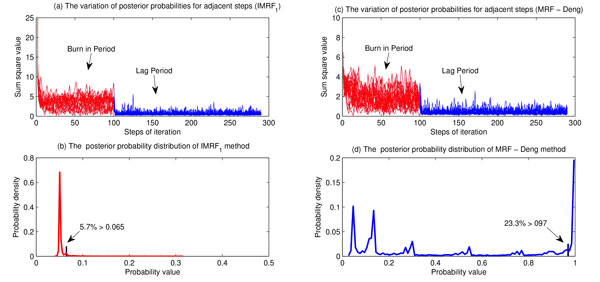
**Analyses of stability and reliability of MRF methods (by using single HPRD PPI network for endocrine disease class)**. (a) The variation of posterior probabilities for adjacent steps of the IMRF_1 _method. (b) The posterior probability distribution of IMRF_1 _method. There are only 5.7% of unknown vertices are predicted with probability larger than 0.065, which means only a small amount significant vertices are predicted with higher probabilities. (c) The variation of posterior probabilities for adjacent steps of the MRF-Deng method; (d) The posterior probability distribution of MRF-Deng method. There are almost 23.3% of unknown vertices are predicted with probability larger than 0.97, which means too many vertices are predicted with very high probabilities.

However, for disease gene identifications, only dozens of disease genes are available for individual disease classes. The estimated parameters of the MRF-Deng method becomes unreliable. This can be seen by analyzing characters of Figure 1. In a Gibbs sampling process, it stops until all Markov processes and parameters are stabilized. However, stabilized Markov processes and parameters do not indicate they converge to expected results. It is also stabilized if most vertices are labelled with 1. Take the Figure 1 (a) and the Figure 1 (c) for example, the variation of posterior probability distributions by using the MRF-Deng method is smaller than the IMRF_1 _method. It seems the performance of the MRF-Deng method is better. However, if we look at Figure 1 (b) and Figure 1 (d), we find that there are 23.3% vertices with probabilities larger than 0.97. This is commonly unreasonable in practices, since it contains too many false positive predictions. The predictions of the IMRF_1 _is reasonable. Most unknown vertices are ranked with a very low probability by using the IMRF_1 _method. Only 5.7% unknown vertices are ranked with probabilities larger than 0.065, and only a few significant vertices are predicted with higher probabilities.

Here, the variation of posterior probabilities for two adjacent steps is calculated from

(22) $$Q\left(t\right)=\overset{n}{\underset{i=1}{\sum }}{\left(P_{i}\left(t\right)-P_{i}\left(t-1\right)\right)}^{2},$$

where *P_i_*(*t*) is the posterior probability *P *(*X_i _*= 1*| X*_[*−i*]_, *θ*) of *g_i _*obtained in the *t^th ^*iteration.

**Figure 2:**
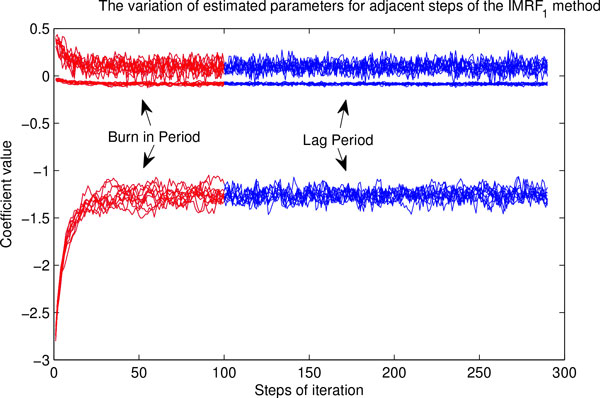
**The variation of estimated parameters for adjacent steps by using the IMRF_1 _method (by using single HPRD PPI network for endocrine disease class)**. There are three coefficients in the model. From top to bottom, they are coefficients of *M*_1_, *M*_0 _and the constant *α*, respectively.

Figure 2 illustrates the variation of estimated parameters for adjacent steps by using the IMRF_1 _method. We can see that all parameters converge very fast, but noises still exist and cannot be reduced by increasing iteration steps. This inspires us to add a "prediction period" for Gibbs sampling processes. The "prediction period" takes the average estimated parameters in the "lag period" as parameters and fixes them hereafter in simulations. The input probabilities of unknown vertices are also obtained by taking the average posterior probabilities in the "lag period".

### Comparisons with the MRF-Deng method

**Figure 3:**
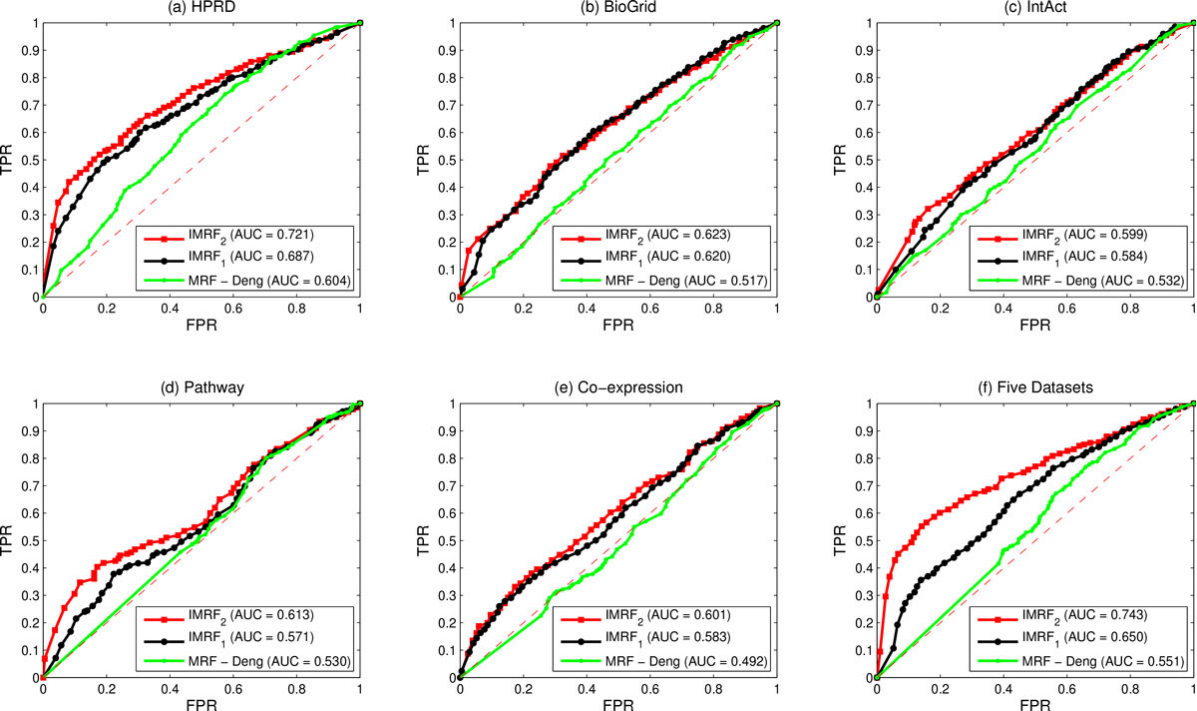
**Comparisons of IMRF_1_, IMRF_2 _and MRF-Deng by using five single biological datasets separately and by integrating them together**. (a) Comparisons by using single HPRD PPI network. (b) Comparisons by using single BioGrid PPI network. (c) Comparisons by using single IntAct PPI network. (d) Comparisons by using single pathway co-exist network. (e) Comparisons by using single gene co-expression network. (f) Comparisons by integrating the above five networks. The red lines are ROC curves by using the IMRF_2 _method. The black lines are ROC curves by using the IMRF_1 _method. The green lines are ROC curves by using the IMRF-Deng method. AUC values are listed in parentheses.

Our improved methods are significantly better than the MRF-Deng method in terms of identifying disease genes. Figure 3 illustrates comparisons of the MRF-Deng method, the IMRF_1 _method and the IMRF_2 _method in terms of ROC curves. Predictions of the IMRF_1 _method is significantly better than that by using the MRF-Deng method, but is a little worse than the IMRF_2 _method, no matter using single biological network or using integrated biological networks. In terms of informativeness of each biological network, the HPRD PPI network (shows in Figure 3 (a)) is the most informative data source, which obtains the highest AUC value in all three methods.

### Integration of heterogeneous data sources

Different biological datasets are commonly heterogeneous. When information in those data is integrated, noises are also integrated. Hence, an inappropriate method may result in a set of worse predictions than using only single dataset. Generally, various data integration methods can be divided into two categories: (1) by using a mixed network and (2) by using several separated networks. Generally, separated networks contain more information than the mixed network, since it is very easy to generate the mixed network from several separated networks but not vice versa. One advantage of the MRF model is that it takes the whole network into consideration, which potentially yields better performance than those using mixed network ones.

**Figure 4:**
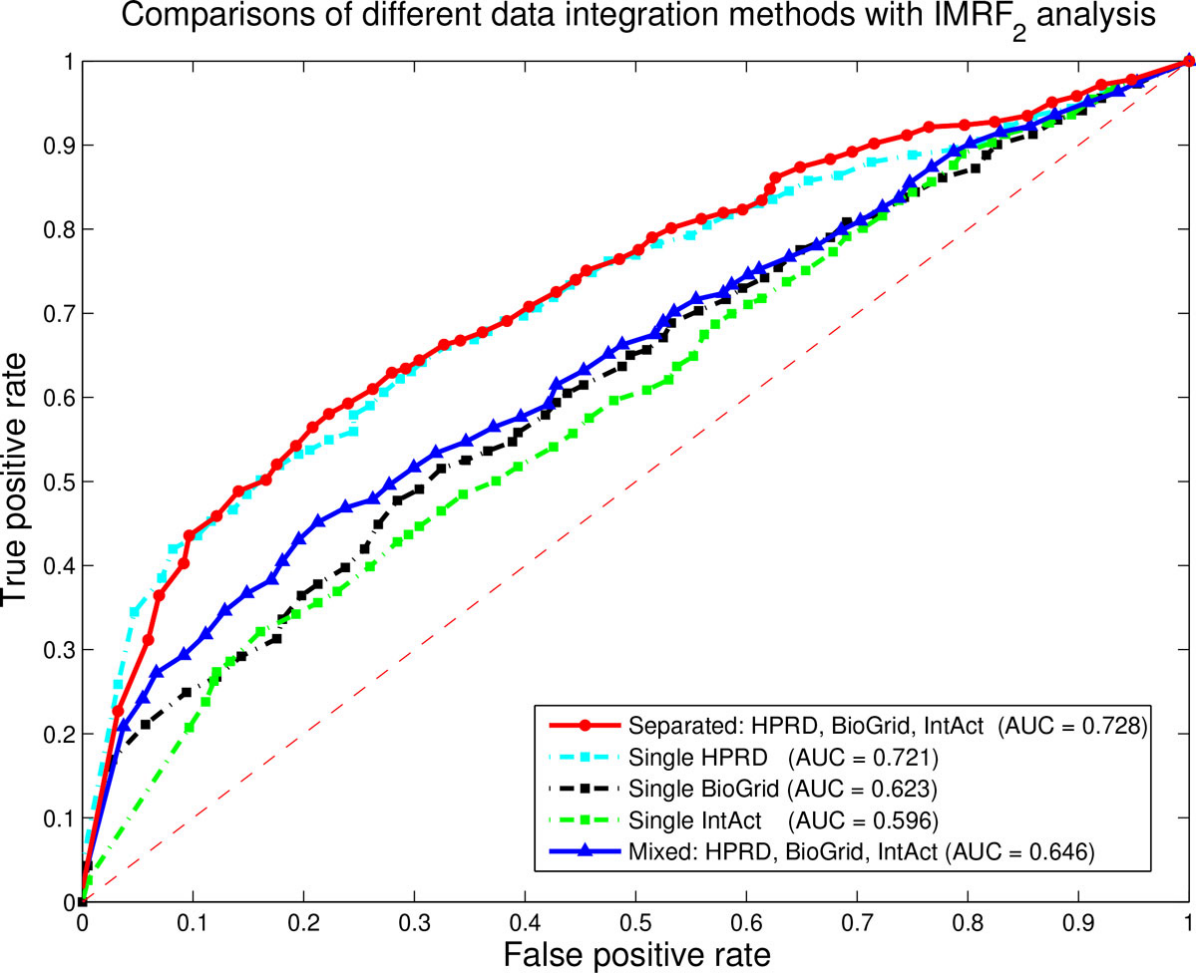
**Comparisons of different data integration methods with IMRF_2 _analysis by using three PPI networks**. The red solid line represents the ROC curve by integrating three PPI networks. The cyan dash-dot line represents the ROC curve by using single HPRD PPI networks. The black dash-dot line represents the ROC curve by using single BioGrid PPI networks. The green dash-dot line represents the ROC curve by using single IntAct PPI networks. The blue solid line represents the ROC curve by using the mixed PPI network. AUC values are listed in parentheses.

In Figures 4, we use the most stable IMRF_2 _method to compare the differences between different kinds of data integration methods. The separated network method achieves the best performance among all predictions, while the mixed network method achieves only modest performance. It seems that the mixed network method combines informations of individual datasets together with their noises, which does not improve its performance by integrating multiple datasets.

### Comparisons by using multiple data sources

**Figure 5:**
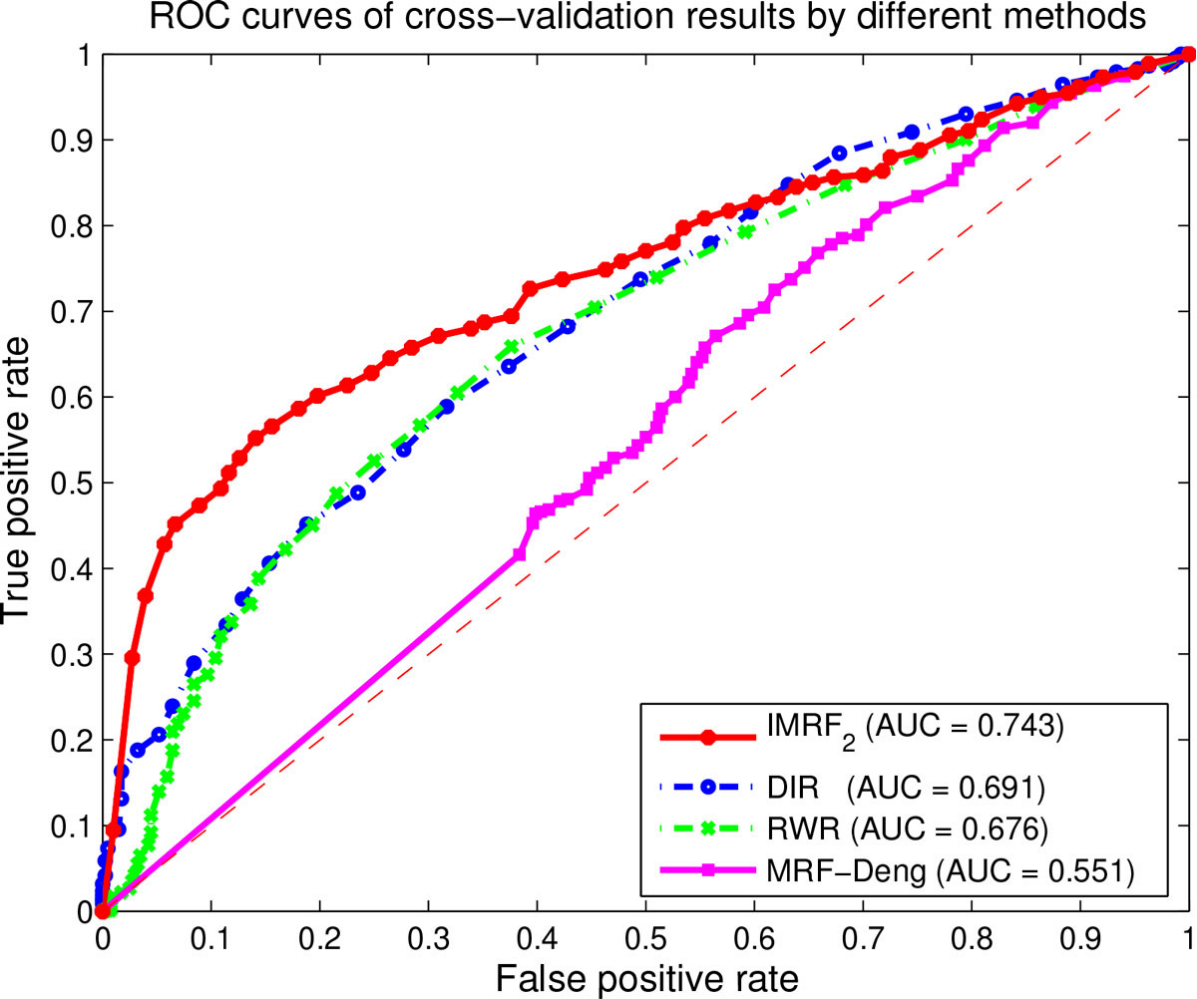
**ROC curves of cross-validation results of different methods by integrating five biological networks**. The red solid line represents the ROC curve by using the IMRF_2 _method. The blue dash-dot line represents the ROC curve by using the DIR method. The green dash-dot line represents the ROC curve by using the RWR method. The Magenta solid line represents the ROC curve by using the MRF-Deng method. AUC values are listed in parentheses.

The IMRF_2 _method is compared with the RWR algorithm, the DIR algorithm and the MRF-Deng algorithm, respectively. Figure 5 illustrates ROC cross-validation results by integrating all five biological networks. The IMRF_2 _method achieves the highest AUC score at 0.743, followed by the DIR algorithm (AUC = 0.691) and the RWR algorithm (AUC = 0.676). The MRF-Deng method achieves the AUC score only at 0.551. It also shows that the separated network interaction method performs better than the mixed network RWR method.

## Conclusions

In this paper, we have presented an improved multiple data integration method for prioritizing human disease genes, which is based on the theory of MRF and the method of Bayesian analysis. The presented method is both flexible in terms of integrating different kinds of biological data and reliable in terms of prioritizing human disease genes. Compared to the MRF-Deng method [30], two strategies have been developed to significantly improve the performance of the MRF method for disease gene identifications.

Firstly, parameters of our improved MRF methods are estimated according to all labelled vertices in integrated biological networks, instead of estimating them according to only known vertices. Moreover, parameters are updated together with sampling labels during iterations, instead of using fixed parameters. The improved parameter estimation method makes our MRF methods more stable and more reliable.

Secondly, a new "prediction period" is added to Gibbs sampling process. Parameters of this period is obtained by taking average parameters in the previous "lag period" and is fixed during iterations of this period. The input probability is also obtained by taking average of posterior probabilities in the "lag period". This strategy significant improves the prediction accuracy of our method.

Predictions when integrating known gene-disease associations, protein complexes, PPIs, pathways and gene expression profiles achieve the AUC score of 0.743, which is better than the RWR method and the DIR method by using the same datasets.

## List of abbreviations

MRF, Markov random field; PPI, protein-protein interaction; RWR, random walk with restart; DIR, data integration rank; MLE, maximum likelihood estimation; MCMC, Markov chain Monte Carlo; OMIM, online Mendelian inheritance in man; PCC, Pearson correlation coefficient; ROC, receiver operating characteristic; AUC, area under the ROC curve.

## Competing interests

The authors declare that they have no competing interests.

## Authors' contributions

FXW and BC initiated this study and designed algorithms and experiments. BC performed the experiments, analyzed the results, and drafted the manuscript. FXW, JXW and ML revised the manuscript. All authors have read and approved the final manuscript.

## Declarations

The publication costs for this article were supported by the Natural Sciences and Engineering Research Council of Canada (NSERC), the National Natural Science Foundation of China under Grant No.61232001 and No. 61370024, and the Program for New Century Excellent Talents in University (NCET-12-0547).

This article has been published as part of *BMC Medical Genomics *Volume 7 Supplement 2, 2014: IEEE International Conference on Bioinformatics and Biomedicine (BIBM 2013): Bioinformatics in Medical Genomics. The full contents of the supplement are available online at http://www.biomedcentral.com/bmcmedgenomics/supplements/7/S2.
